# RNAi-Mediated Silencing of Pgants Shows Core 1 *O-*Glycans Are Required for Pupation in *Tribolium castaneum*

**DOI:** 10.3389/fphys.2021.629682

**Published:** 2021-03-24

**Authors:** Weidong Li, Kristof De Schutter, Els J. M. Van Damme, Guy Smagghe

**Affiliations:** ^1^Laboratory of Agrozoology, Department of Plants and Crops, Faculty of Bioscience Engineering, Ghent University, Ghent, Belgium; ^2^Laboratory of Biochemistry and Glycobiology, Department of Biotechnology, Faculty of Bioscience Engineering, Ghent University, Ghent, Belgium

**Keywords:** *O*-glycosylation, oligosaccharide, gene expression, RNAi, *Tribolium castaneum*, metamorphosis

## Abstract

Protein glycosylation is one of the most common and most important post-translational modifications. Despite the growing knowledge on *N-*glycosylation, the research on *O-*glycosylation is lagging behind. This study investigates the importance of *O-*glycosylation in the post-embryonic development of insects using the red flour beetle, *Tribolium castaneum*, as a model. We identified 28 *O-*glycosylation-related genes (OGRGs) in the genome of the red flour beetle. 14 OGRGs were selected for functional analysis based on their involvement in the initial attachment of the carbohydrate in the different *O-*glycosylation pathways or the further elongation of the most abundant *O-*glycans and, in addition, showing severe RNAi-induced phenotypes in *Drosophila melanogaster*. The expression profile of these OGRGs was mapped throughout the developmental stages of the insect and in the different tissues of the pupa and adult. Subsequently, these genes were silenced using RNA interference (RNAi) to analyze their role in development. A broad spectrum of phenotypes was observed: from subtle effects and disrupted wing formation when silencing the genes involved in *O-*mannosylation, to blockage of pupation and high mortality after silencing of the genes involved in *O*-GalNAc and core 1 *O*-glycan (*O*-GalNAc-Gal) synthesis. RNAi experiments were also performed to assess the effects of blocking multiple pathways of *O*-glycosylation. However, the observed phenotypes induced by multiple RNAi were similar to those of the single gene RNAi experiments. The silencing of OGRGs often resulted in high mortality and wing phenotypes, indicating the importance of *O*-glycosylation for the survival of the insect and the formation of wings during the post-embryonic development of *T. castaneum*.

## Introduction

**FIGURE 1:**
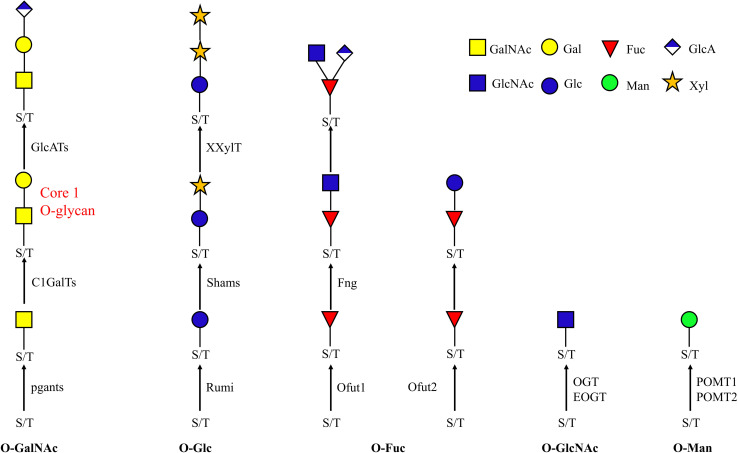
O-glycan biosynthesis pathway. *O-*glycans are attached to serine and threonine. The known enzymes responsible for each attachment are indicated beside the arrow. Ofut1 is responsible for transferring monosaccharide specifically to EGF repeats; Ofut2 specifically transfers Fuc to TSRs. EOGT and Rumi specifically transfer sugars to EGF repeats. POMT1 and POMT2 cooperate in the transfer of Man. *pgants*, N-acetylgalactosaminyltransferases; *POMTs*, protein *O-*mannosyltransferases; *Ofuts*, *O-*fucosyltransferases; *OGTs*, *O-*GlcNAc transferases; *C1GalT*, core 1 β1,3-galactosyltransferase; *fng*, Fuc-specific *O-*GlcNAc transferase; *Rumi*, protein *O-*glucosyltransferase; GalNAc, N-acetylgalactosamine; Gal, galactose; GlcNAc, N-acetylglucosamine; Fuc, fucose; Man, mannose; Glc, glucose; GlcA, glucuronic acid; Xyl, xylose.

The decoration of proteins with carbohydrates is one of the most important post-translational modifications. One of the major types is *O*-glycosylation, in which a sugar is transferred to the oxygen atom of a hydroxyl group of mainly serine and threonine, but in some cases hydroxyproline (plant), hydroxylysine (collagens) or tyrosine (prokaryotes) residues can also be *O*-glycosylated (Spiro et al., 1971; Kieliszewski, 2001; Zarschler et al., 2010). *O-*glycosylation is found in all kingdoms of life, including mammals, insects, fungi, archaea, plants and bacteria (Ten Hagen, 2015; Eichler and Koomey, 2017). *O-*glycosylation plays a crucial role in many biological processes, including, stress response and immunization through cell-to-cell interaction and protein-to-protein interaction, and influences protein stability, folding, localization and secretion (Fukuda, 2002; Schjoldager et al., 2011; Ashwood et al., 2017; Chaffey et al., 2017a, b; Martinez et al., 2017). Previous studies have shown that unusual *O-*glycosylation is associated with a variety of diseases and cancers (Yang et al., 2017). In contrast to *N-*glycosylation, where an oligosaccharide (glycan precursor) is transferred to the nascent protein in the endoplasmic reticulum (ER), *O-*glycans are synthesized by the stepwise addition of monosaccharides in the ER, Golgi or cytosol (Spiro, 2002; De Pourcq et al., 2010; Dell et al., 2010; Figure 1). In insects, *O-*glycans consist mainly of eight monosaccharides –N-acetylgalactosamine (GalNAc), galactose (Gal), N-acetylglucosamine (GlcNAc), fucose (Fuc), mannose (Man), glucose (Glc), glucuronic acid (GlcA) and xylose (Xyl)– which can be assembled into oligosaccharides or polysaccharides. The attached residue in *O-*glycosylation can be GalNAc, GlcNAc, Fuc, Man, Glc, Xyl (Haltom and Jafar-Nejad, 2015; Walski et al., 2017), while the attached monosaccharide residue in *N-*glycosylation is always a GlcNAc. This heterogeneity in the attachment residue makes *O-*glycosylation more diverse and complex than *N-*glycosylation. This complexity makes the study of *O-*glycosylation in insects challenging. So far there are only a couple of studies focused on *O-*glycosylation in insects such as *Drosophila melanogaster* (Aoki et al., 2008), mosquito (Kurz et al., 2015) and silkworm (Soya et al., 2016). These studies investigated the *O-*glycan profile during specific developmental stages (embryos, larvae) or in specific tissues (nervous system). In *Drosophila* embryos, the *O-*GalNAc-Gal (core 1 structure or T antigen) is the most prevalent *O-*glycan structure representing 55% of the total *O-*glycan pool; *O-*GalNAc (Tn antigen) and *O-*GlcNAc account for 19% of the total profile; *O-*GalNAc-Gal-GlcA and *O-*Fuc(GlcNAc)-GlcA occupy about 10% each; while *O-*GalNAc(Gal)-GlcA, *O-*Man/*O-*Glc, and *O-*GalNAc(HexNAc)-GlcA each account for around 1% of the total *O-*glycan profile (HexNAc, N-acetylhexosamine, including GalNAc and GlcNAc). Other *O-*glycans are much less abundant (Aoki et al., 2008). Although the *O-*glycan profiles of a specific tissue or at a specific stage yield valuable information, they do not provide a general understanding of *O-*glycosylation in the insect.

*O-*glycosylation involves several different pathways, leading to the attachment of *O-*GalNAc, *O-*GlcNAc, *O-*Fuc, *O-*Man, *O-*Glc type *O-*glycans and the OGRGs encode the enzymes responsible for the synthesis of these diverse *O-*glycan structures (Figure 1). In *Drosophila*, 12 polypeptide N-acetylgalactosaminyltransferases (pgants) are responsible for transferring GalNAc to protein substrates, and 8 core 1 β1,3-galactosyltransferases (C1GalTs) are responsible for transferring Gal to the *O-*linked GalNAc. These pgant genes are characterized by a distinct spatial and temporal expression pattern during development (Tian and Hagen, 2006), for example, *pgant3* is located in the wing disk and is responsible for wing adhesion, loss of *pgant3* causes wing blistering in *Drosophila* (Zhang et al., 2008, 2010). *Pgant5* and *pgant35A* have an influence on gut acidification and the respiratory system, respectively (Tran et al., 2012) and mutations or silencing of these genes causes complete lethality in the fruit fly (Yamamoto-Hino et al., 2015). In *D. melanogaster*, many proteins were shown to carry mucin-type *O-*glycans (Schwientek et al., 2007; Zhang et al., 2008; Walski et al., 2017) and knockdown of these proteins resulted in lethality (Mummery-Widmer et al., 2009; Fraichard et al., 2010; Kong et al., 2010; Maynard et al., 2010; Neely et al., 2010; Nagarkar-Jaiswal et al., 2015). The *O*-GlcNAc transferases OGT is responsible for transferring GlcNAc to intracellular target proteins (Ogawa et al., 2015), while EOGT specifically glycosylates Notch and its ligands (Li et al., 2020), and interacts with Notch signaling, required for many developmental processes (Müller et al., 2013). *O-*mannosylation of Dystroglycan (a major non-integrin extracellular matrix receptor) occurs through the cooperation of the protein *O*-mannosyltransferases (POMT) *POMT1* and *POMT2* (Ichimiya et al., 2004; Nakamura et al., 2010). Since this protein is required for proper muscle formation, its mutation caused major mortality and locomotor behavior defective phenotypes in *Drosophila* (Shcherbata et al., 2007; Yatsenko et al., 2014). In *Drosophila*, Ofut1 is an *O-*fucosyltransferase (Ofut) specific for EGF repeats while *Ofut2* transfers Fuc to thrombospondin type-1 repeats (TSRs) (Luo et al., 2006). The Fucose-specific *O-*GlcNAc transferase (*Fng*) encodes a glycosyltransferase responsible for transferring GlcNAc specifically to the *O-*fucose of EGF repeats (Matsumoto et al., 2015), and mutation of the gene in fruit fly is lethal. *O*-Glc structures represent less than 1% of the identified *O*-glycans in *Drosophila* embryos. However, in silkworms, monosaccharide profiling revealed that Glc is the most abundant monosaccharide in the nervous system during development (Aoki et al., 2008; Soya et al., 2016). Insects encode one glucosyltransferase, Rumi, which catalyzes the transfer of Glc residues to EGF repeats of secreted proteins and plays a redundant role with Ofut1 in the trafficking of Notch out of the ER and in the activation of Notch signaling (Ishio et al., 2015; Matsumoto et al., 2016).

In *D. melanogaster* RNAi of these OGRGs indicated that *O-*glycosylation was essential for viability and movement, and plays vital roles in the respiratory, immune and nervous system (Ichimiya et al., 2004; Okajima et al., 2005; Tian and Hagen, 2006; Zhang et al., 2008; Sinclair et al., 2009; Sanders et al., 2010; Zhang et al., 2010; Park et al., 2011; Haltom and Jafar-Nejad, 2015; Ten Hagen, 2015). However, the specific function of *O-*glycosylation in the development of insects remains unclear.

This project addressed the importance of *O-*glycosylation in the post-embryonic development of the holometabolic model insect, *Tribolium castaneum* or red flour beetle. Therefore, we first identified the genes encoding enzymes involved in *O-*glycosylation in the genome of *T. castaneum* and analyzed the changes in transcription levels of the OGRGs involved in the synthesis of the main *O-*glycan structures and their precursor monosaccharides throughout the different life stages and body parts of the red flour beetle. These genes include the UDP-Galactose 4-epimerase (GALE, an enzyme catalyzing the interconversion of UDP-Gal and UDP-Glc in the final step of the Leloir pathway of galactose metabolism, and also the interconversion of UDP-GalNAc and UDP-GlcNAc), and the OGRGs involved in the first two steps of the different *O*-glycosylation pathways (*pgants*, *POMTs*, *Ofuts*, *OGTs*, *C1GalT*, *fng* and *Rumi*). In addition, the role of *O-*glycosylation in development was studied using RNAi of the OGRGs by injecting dsRNA in the larval stage. We discovered that most *O-*glycan transferases reached a peak of gene expression in the pupal stage, and a decrease in OGRG transcription levels resulted in mortality as well as abnormal wings and antennae. Therefore, we hypothesized that *O-*glycosylation plays an important role in metamorphosis and wing development of *T. castaneum*.

## Materials and Methods

### Insect Culture

The wild type strain *T. castaneum* GA-1 was used in all experiments. Insects were reared on whole wheat flour containing 5% yeast powder as daily diet under standard conditions (30°C and humidity 60% in darkness) as previously described (Walski et al., 2016).

### Identification of OGRGs and Phylogenetic Analyses

Protein sequences encoding the OGRGs from *D. melanogaster* were used to perform BLAST searches against the *T. castaneum* genome through *Tribolium* BLAST^1^ (*E*-value < 0.0001). Protein sequences were downloaded from^2^ and screened for the presence of conserved protein domains using interproscan 5 (Jones et al., 2014). The program was downloaded^3^ and locally installed. Only sequences with the required domains were retained. Phylogenetic trees were constructed including known sequences from *D. melanogaster*, *Leptinotarsa decemlineata*, *Aedes aegypti*, *Bombus terrestris*, *Bombyx mori*, *Nilaparvata lugens*, *Acyrthosyphon pisum* and *Homo sampiens* in MEGA X using Maximum Likelihood method. The ML substitution matrix was analyzed and the best fitting model was selected based on the BIC value. The phylogeny was tested with 1000 bootstrap replicates. Protein sequences from all species were identified in BLAST searches against the respective genomes available from NCBI^4^ (*E*-value < 0.0001). All identified OGRGs of *T. castaneum* are listed in Table 1. All protein sequences used in the phylogenetic analysis were collected in Supplementary File 1.

**TABLE 1:** Overview of putative genes involved in *O-*glycosylation of *T. castaneum*, included in this study.

*Drosophila* gene	NCBI reference	*Tribolium* orthologs	In this paper	Function
*pgant3*	NP_610256.1	TC005702	*Tcpgant3*	Polypeptide GalNAc transferase
*pgant5*	NP_608906.2	TC005902	*Tcpgant5*	Polypeptide GalNAc transferase
*pgant35A*	NP_652069.2	TC013148	*Tcpgant35A*	Polypeptide GalNAc transferase
*C1GalTA*	NP_723427.1	TC030587	*TcC1GalTA*	Core 1 Galactosyltransferase
*OGT (sxc)*	NP_724406.1	TC003916	*TcOGT*	*O-*GlcNAc transferase
*EOGT*	NP_608678.1	TC013339	*TcEOGT*	EGF domain-specific *O-*GlcNAc transferase
*Ofut1*	NP_610931.1	TC013385	*TcOfut1*	*O-*fucosyltransferase
*Ofut2*	NP_569916.1	TC030583	*TcOfut2*	*O-*fucosyltransferase
*fng(fringe)*	NP_524191.1	TC011785	*Tcfng*	Fucose-specific *O-*GlcNAc transferase
*POMT1 (rt)*	Q9VTK2.2	TC010429	*TcPOMT1*	Protein *O-*mannosyltransferase
*POMT2 (tw)*	NP_569858.1	TC033711	*TcPOMT2*	Protein *O-*mannosyltransferase
*Rumi*	AAN13920.1	TC013552	*TcRumi*	Protein *O-*glucosyltransferase
*GALE*	AAF47398.1	TC009598	*TcGALE1*	UDP-galactose-4-epimerase
		TC009301	*TcGALE2*	

### RNA Extraction and cDNA Synthesis

Samples were collected from egg to adult stage of *T. castaneum.* First, eggs that were laid within a period of 24 h were collected using an 800 Mic sieve. After hatching, insects were collected every three days for second to sixth instar and prepupa. Then samples of pupa and late pupa were collected at 20 and 22 days post hatching. Insects collected at 24 and 27 days post hatching represent the early adult and mature adult samples, respectively. Ten milligrams of fresh insects were taken as a sample for RNA extraction. Additionally, head, thorax, abdomen, wings (forewings and hindwings) and legs were dissected under a microscope, and then separately collected from 10 pupae or 20 adults. Total RNA was extracted using the RNeasy mini kit (Qiagen) according to the manufacturer’s instructions. The concentration of total RNA was measured by Nanodrop ND-1000 (Thermo Scientific) and the quantity was checked on a 1.5% agarose gel after removing genomic DNA using the TURBO DNA-free^TM^ Kit (Ambion/Life Technologies). cDNA synthesis was conducted following the manufacturer’s protocol using SuperScript^TM^ II Reverse Transcriptase (Invitrogen/Life Technologies). One microgram of total RNA was used in cDNA synthesis with an Oligo(dT)_12_ primer. Finally, the cDNA solution was stored at −20°C for subsequent experiments.

### Primers

All the primers were designed by Primer3Plus^5^. The sequences of the primers for the reference genes were obtained from publications (Lord et al., 2010; Toutges et al., 2010; Vuerinckx et al., 2011; Feliciello et al., 2015). Primer specificity was checked using PrimerBlast in NCBI and on gel. All primer sequences are listed in the Supplementary Tables 1, 2.

### Reverse Transcription Quantitative PCR

Reverse transcription quantitative PCR (RT-qPCR) was performed using a CFX96TM Real-Time PCR Detection system (BioRad) with GoTaq^®^ qPCR Master Mix (Promega). A standard curve with a serial dilution of cDNA was made for each primer pair to determine the amplification efficiency, followed by an analysis of the melting curve to ensure specificity. Each qPCR reaction was performed in duplicate, which contained 10 μl of GoTaq^®^ qPCR Master Mix, 0.4 μl of 10 μM forward primer, 0.4 μl of 10 μM reverse primer (Invitrogen), 8.2 μl of water and 1 μl of cDNA. All reactions were performed with 95°C for 5 min, followed by 40 cycles of 95°C for 15 s, 60°C for 60 s. No-template controls and no-reverse transcriptase controls were performed to exclude contamination. Ubiquitin and RPS18 were chosen as reference genes based on previous studies and our optimization with qBase + software (Biogazelle). All the gene expressions were analyzed by qBase + software (Biogazelle).

### Larva RNAi Experiments

DsRNAs were synthesized following the instructions of the MEGAscript^®^ RNAi Kit (Life Technologies) with primers containing the T7 promoter sequence. The concentration of dsRNA was measured by Nanodrop ND-1000 (Thermo Scientific). The quality was checked on a 1.5% agarose gel. Fourth-instar larvae (with an individual fresh weight of 1.2 ± 0.2 mg) were injected with approximately 200 nl of dsRNA at 1 μg/μl using a FemtoJet^®^ injector (Eppendorf) after 4 min diethyl ether-anesthetization. Insects in the control group were injected with an equal amount of dsGFP (200 ng) in the same volume. About 60 larvae were injected for each gene in two biological replicates. In the double and triple RNAi experiments, fourth-instar larvae were injected with the same amount of dsRNA for each gene as in the single RNAi experiments.

After 3 h of recovery, the insects that died because of injection or physical injury were discarded. Larvae were kept in an incubator under standard conditions and checked every second day for mutant phenotype, mortality, time of pupation as well as time of adult eclosion. Images were taken for each insect using a Leica SD6 stereomicroscope. Elytra length and elytra gap of pupa was measured in Fiji software^6^. RNAi efficiency was inspected by RT-qPCR at larva [6 days post-injection (dpi)], pupa (11 dpi) and adult (16 dpi) stage. For measurement of adult motility at 20 dpi, adults were put in the middle of a square cardboard box and filmed for at least 30 s using a digital camera after a short adaption period. The recorded videos were transformed into images at one-second intervals using Free Video to JPG Converter. Then adult motility was measured in Fiji software. In brief, images were combined to the extended depth of field pictures as previously optimized (Walski et al., 2016) and measured using a segmented line. Data analysis was performed using independent *t*-tests in SPSS 23.

## Results

### Expression of Putative Genes Related to *O*-Glycosylation During Development

*O-*glycans are generated through an extended set of enzymes encoded by OGRGs. The identification of these genes and their expression patterns throughout post-embryonic development could shed light on the role of *O-*glycosylation during insect development. OGRGs in the genome of *T. castaneum* were identified through BLAST searches. Using the protein sequences for the OGRGs from *D. melanogaster* (Adams et al., 2000) as a query, a total of 28 putative OGRG sequences were identified in the red flour beetle genome (Supplementary Table 4). Phylogenetic trees were constructed to confirm the true orthology of the identified sequences (Supplementary Figure 1). Overall, the identified *Tribolium* sequences show close homology with the orthologs from *L. decemlineata*. Similarly, the dipterian (*D. melanogaster* and *A. aegypti*) and hemipteran (*N. lugens* and *A. pisum*) sequences cluster together in most of the trees. Remarkable was the divergence of the coleopteran Rumi cluster from the other insects, forming a separate branch in the phylogenetic tree (Supplementary Figure 1F). The clustering of pgants 2, 5, 7, and 9 suggest an ancestral evolution, with the former three even including human orthologs. In contrast, the orthologs of pgants 4, 6, 8, and 10 group in one cluster and suggest a more species specific development (Supplementary Figure 1G).

**FIGURE 2:**
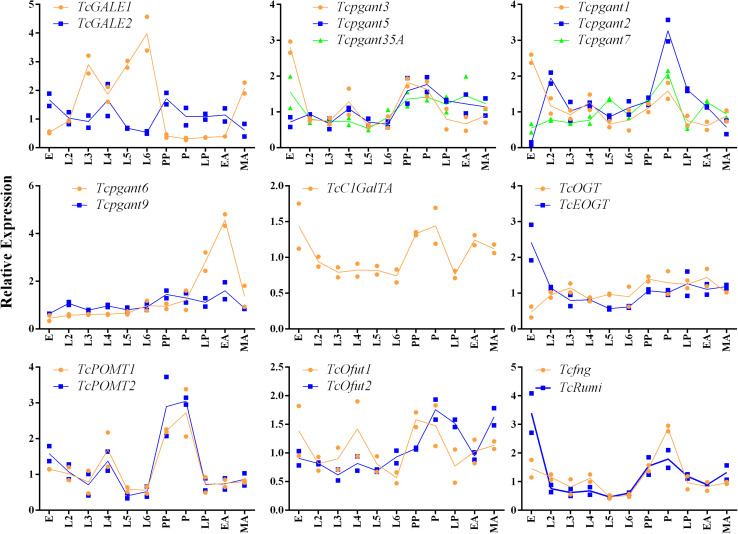
Relative expression of the OGRGs during development of *Tribolium castaneum.* Eggs samples (E) were collected within a period of 24 h. second to sixth-instar larvae (L2-L6) and prepupae (PP) were collected every three days after egg-hatching. At 20 and 22 days post egg-hatching, samples of pupa (P) and late pupa (LP) were collected. Early adult (EA) and mature adult (MA) insects were collected at 24 and 27 days post egg-hatching. Relative gene expression was measured by RT-qPCR and normalized using *TcUbiquitin* and *TcRPS18* as reference genes. Lines connect the mean values of two biological replicates.

Based on their position in the *O-*glycosylation pathway, their involvement in the synthesis of the most abundant *O-*glycans or with orthologs showing clear mutant phenotypes in *D. melanogaster*, 19 OGRGs (Table 1) were selected for an in-depth analysis of their expression patterns (Aoki et al., 2008; Ten Hagen et al., 2009; Haltom and Jafar-Nejad, 2015). Transcription levels were quantified for the different developmental stages from egg to adult insects (Figure 2). Statistically significant differences were observed in the transcription levels for all genes (ANOVA, *P* < 0.05). Most of the OGRGs showed an increase in expression during the pupal stages (four in prepupa, ten in pupa and one in late pupa) (Figure 2). In contrast, mRNA levels of *TcGALE1* were high during the larval stages, with the peak of expression at the sixth-instar larval stage, and a decrease during the pupal stage and early adult stage. *Tcpgant6* transcription levels are low during the larval and early pupal stages, and increase during the late pupal stage, reaching a peak at the early adult stage. Several genes showed high transcription levels at the egg stage, with 5 genes showing their peak expression at this stage. While the expression profiles for genes coding for paralogous enzymes mostly showed similar patterns (*pgants*, *POMTs*, *Ofuts*, and *OGTs*), the expression profiles for *TcGALE1* and *TcGALE2* showed opposite patterns (Figure 2).

**FIGURE 3:**
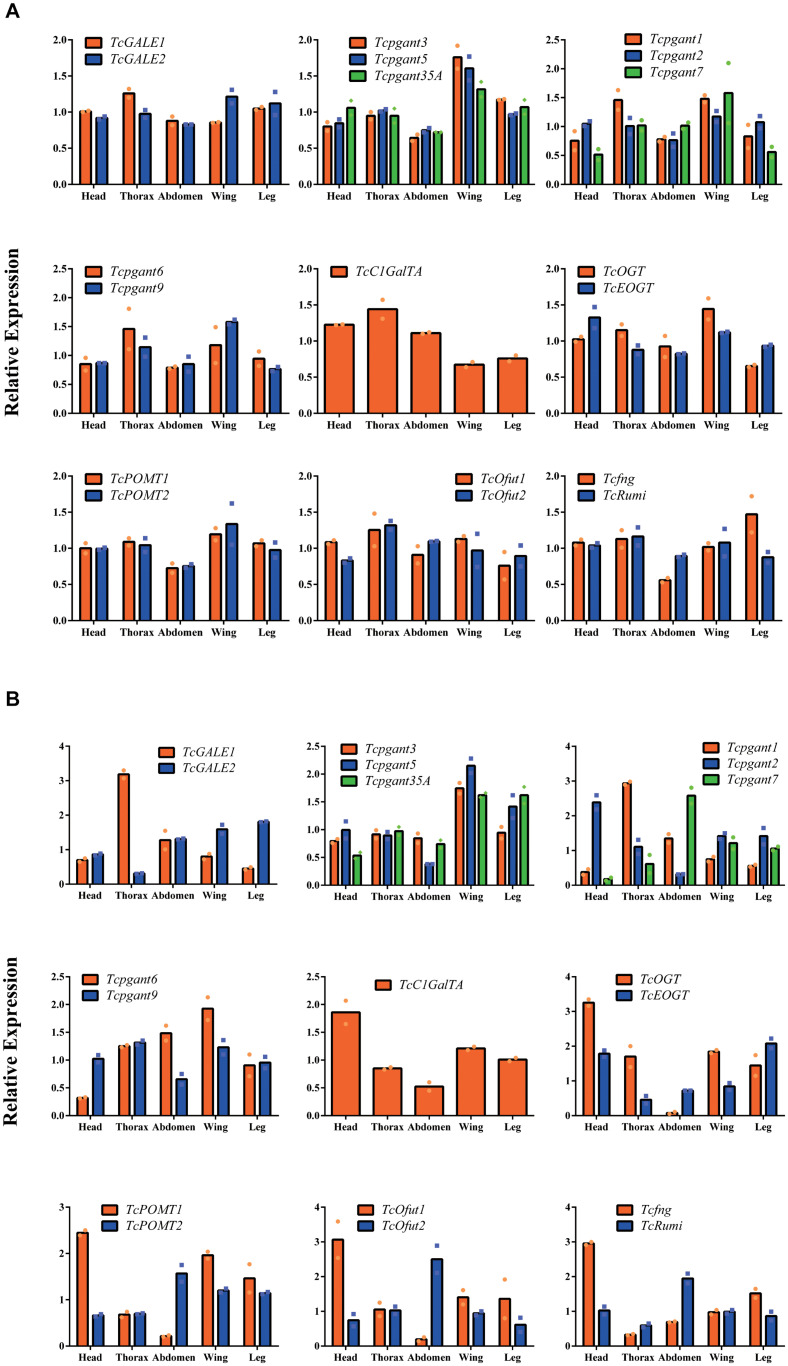
Tissue specificity of the OGRGs at pupa and adult stage of *Tribolium castaneum*. OGRGs expression in head, thorax, abdomen, wings (forewings and hindwings) and legs was examined at the pupa and adult stage of the red flour beetle. The different tissues were dissected under a microscope, and separately collected from 10 pupae or 20 adults. Relative gene expression was measured by RT-qPCR and normalized to reference genes (*TcUbiquitin* and *TcRPS18*). The bars represent the mean values of two biological replicates. **(A)** The tissue specificity of OGRGs at the pupal stage of the red flour beetle. **(B)** The tissue specificity of OGRGs at the adult stage.

To further characterize the expression profile of the OGRGs in the red flour beetle, the transcription levels for these genes were analyzed in different tissues (head, thorax, abdomen, wings (forewings and hindwings) and legs) at the pupal and adult stage. The results showed that during the pupal stage *TcGALE2*, *Tcpgant1*, *Tcpgant3*, *Tcpgant5*, *Tcpgant7*, *Tcpgant9*, *Tcpgant35A*, *TcOGT*, *TcOfut1*, *TcPOMT1*, *TcPOMT2*, *Tcfng*, and *TcRumi* are highly expressed in the wings (Figure 3A). In addition, *Tcpgant6*, *TcGALE1*, *TcC1GalTA*, *TcOfut1*,*TcOfut2*, and *TcRumi* exhibited their highest expression in the thorax. Interestingly, the unique gene showing topmost expression in the legs is *Tcfng*. Except for *Tcpgant7*, *TcC1GalTA* and *TcOfut2*, most genes showed a lower expression in the abdomen during the pupal stage (Figure 3A). As observed in the temporal expression profile, most paralogous enzymes (*pgants*, *POMTs*, *Ofuts*, and *OGTs*) showed a similar pattern over the different tissues of the pupa; except for the GALEs that show opposite patterns. Moreover, *POMTs*, *Ofuts* and *OGTs* are nearly constitutively expressed in all parts of the pupal body (Figure 3A). At the adult stage, the tissue specificity of some genes changed (Figure 3B). The peak in *Tcpgant7* expression shifted from the wings in the pupal stage to the abdomen in the adult stage. Also for *TcRumi*, a shift in expression to the abdomen was observed. Similarly, *Tcpgant1* expression became dominant in the thorax and decreased in the wings. Expression of *Tcpgant2* peaked in the head at the adult stage. *GALE2*, *Tcpgant3*, *Tcpgant5*, *Tcpgant6*, *Tcpgant9*, *Tcpgant35A*, *TcC1GalTA*, *TcOGT*, *TcOfut1*, *TcPOMT1* and *TcPOMT2* were highly expressed in the wings. In the abdomen, *Tcpgant7*, *TcOfut2*, *TcPOMT2*, and *TcRumi* showed the highest expression, while *Tcpgant2*, *Tcpgant5*, *Tcpgant9*, *TcC1GalTA*, *TcOGT*, *TcOfut1* and *TcPOMT1* showed their lowest expression levels (Figure 3B). In addition, *Tcpgant2*, *TcC1GalTA*, *TcOGT*, *TcOfut1*, *TcPOMT1*, *Tcfng* showed their peak expression in the head. *TcGALE2* and *TcEOGT* were expressed at the highest level in the legs. *Tcpgant1* and *TcGALE1* showed the highest expression in the thorax. Although their spatial expression pattern was similar in the pupa, *OGTs*, *Ofuts* and *POMTs* showed an inverse expression pattern at the adult stage (Figure 3B). The results of the expression analysis indicated that several pgant genes have distinct temporal and spatial expression patterns (Figures 2, 3).

### Silencing of Genes Related to Core 1 *O*-Glycan Synthesis Affects Pupal Development

**FIGURE 4:**
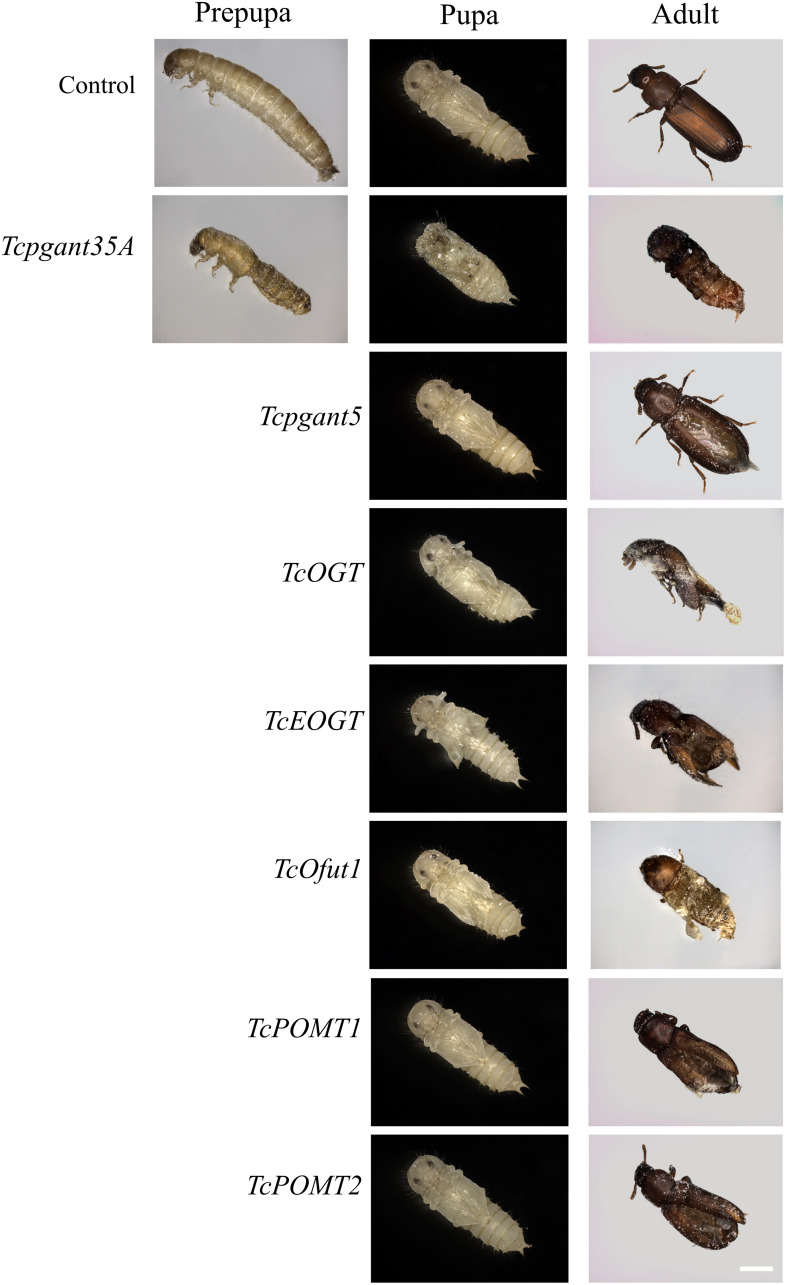
RNAi phenotypes of single RNAi experiments. RNAi of *Tcpgant5* affected elytra development; RNAi of *Tcpgant35A* caused severe phenotypes and prevented pupation as well as eclosion. RNAi of *TcOGT* and *TcEOGT* caused strong phenotypes. RNAi of *TcPOMT1* and *TcPOMT2* resulted in adult wing malformation. RNAi of *TcOfut1* destroyed elytra development. Scale bar is 1 mm.

**FIGURE 5:**
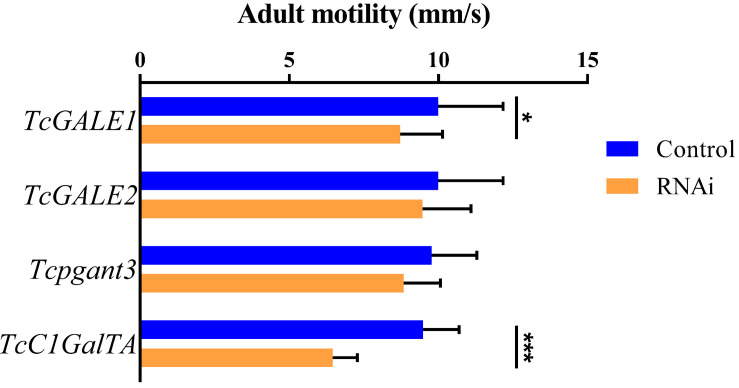
RNAi effects on adult motility. RNAi of *TcC1GalTA* decreased adult mobility (*n* = 28, 38, 51, 57, from top to down). Error bars represent SEM of two biological replicates, *P < 0.05, **P < 0.01, ***P < 0.001.

The core 1 *O-*glycan structure is the most prevalent *O-*glycan structure in insects and consists of a single GalNAc extended with a single Gal residue. These sugar moieties are transferred to the protein through the sequential action of pgants and core 1 β1,3-galactosyltransferases (C1GalTs) (Figure 1). DsRNA injection targeting the selected genes resulted in an efficient silencing of gene transcription (Supplementary Figure 2). As shown in Figure 4, RNAi of *Tcpgant3* decreased adult mobility by 10% (*P* = 0.011). Silencing of *Tcpgant5* caused a 33% larger pupa elytra gap compared to control (*P* = 0.007), and malformed adult elytra (Figure 4). RNAi of *Tcpgant35A* prevented pupation and eclosion, and yielded a reduction in pupa elytra length (13%, *P* < 0.001) and an increased elytra gap (67%, *P* = 0.045). About 20% of the injected insects died during the larval stages, 40% of the insects died during the transition from larva to pupa, 20% died during the transition from pupa to adult, and in total the mortality was about 90%. Pupae were malformed, and the dead insects showed appearance with adult thorax and pupa abdomen (Figure 4). RNAi of *TcC1GalTA* impaired adult motility (32%, *P* < 0.001) (Figure 5).

### *O*-GlcNAc Glycan Is Required for Pupa and Adult Development

The *Tribolium* genome contains two genes encoding *O-*GlcNAc transferases potentially involved in *O-*GlcNAcylation (Figure 1): *OGT*, transferring GlcNAc to common *O-*glycosylation sites of the peptide, and *EOGT*, which specifically transfers GlcNAc moieties to *O-*glycosylation sites in the EGF domain of secreted and membrane proteins (Varshney and Stanley, 2017). After injection of 4^th^-instar larvae with specific dsRNAs targeting either *TcOGT* or *TcEOGT*, the transcription levels of these genes were reduced by 70% (Supplementary Figure 2). Silencing of either of these genes caused conspicuous phenotypes (Figure 4) and adult mortality (Table 2). Silencing of *TcEOGT* resulted in antenna misorientation, a larger elytra gap compared to the control group (57%, *P* = 0.014), and wing blistering at pupal stage in 5% of the insects (Figure 4). The wings of adults were not closed, and the molting was not completely finished. Upon injection of dsRNAs targeting *TcOGT*, a misorientation of the antennas was observed in the pupa and the adult insects (Figure 4). The elytra of the pupa showed a reduction in size by 10% (*P* < 0.001) and a 5-fold increase in the gap distance between the pupal elytra (*P* < 0.001) (Figure 4) as shown in Supplementary Table 3. As was observed for *TcEOGT* silencing, silencing of *TcOGT* resulted in the inability to close the wings in the adult insects.

**TABLE 2:** Summary of phenotypes caused by larval RNAi.

Gene	Mortality%			No. of injected insects	Phenotype
	Larva	Pupa	Adult		
*Tcpgant3*	1.7	0.0	0.0	47	Adult motility reduced
*Tcpgant5*	3.3	0.0	26.7	58	Pupal elytra apart Adult elytra malformed
*Tcpgant35A*	60.0^a^	5.0	25.0^b^	53	Pupal elytra apart and shorter Pupa malformed Pupation and eclosion blocked
*TcC1GalTA*	1.7	1.7	0.0	60	Adult motility reduced
*TcOGT*	5.0	6.7	66.7	60	Pupal elytra apart and shorter Antenna misorientation Adult wing apart Adult body bent
*TcEOGT*	0.0	0.0	31.7	59	Pupal and adult elytra apart Pupal elytra blister Adult body bent Antenna misorientation
*TcOfut1*	1.7	5.3	75.5	52	Pupal elytra apart and shorter Pupa abnormal Adult elytra malformed
*TcOfut2*	4.7	2.9	0.0	51	None
*Tcfng*	6.7	0.0	0.0	50	None
*TcPOMT1*	7.9	1.7	52.5	45	Pupal elytra shorter Adult elytra malformed
*TcPOMT2*	5.7	1.7	61.8	43	Pupal elytra shorter Adult elytra malformed
*TcRumi*	0.0	1.7	0.0	60	None
*TcGALE1*	7.5	0.0	3.3	52	Adult motility reduced
*TcGALE2*	6.8	0.0	0.0	58	None

*^*a*^Majority of insects died during transition from larva to pupa. ^*b*^Majority of insects died during the transition from pupa to adult.*

### Decreased Expression of *O*-Mannosylation Genes Affects Adult Wing Formation

Insects encode two protein *O-*mannosyltransferases which cooperate to the transfer of Man residues to proteins: *POMT1* and *POMT2* (Figure 1). Silencing of either of these two genes in *T. castaneum* larvae caused abnormal elytra (Figure 4) and adult mortality (Table 2). The length of the pupal elytra was found to be significantly reduced (*P* < 0.001) after injection of dsRNAs targeting *TcPOMT1* and *TcPOMT2* (10% and 9%, respectively) compared to the control treatment. In addition, adult wings were malformed and not closed. Silencing of these genes did not have an impact on the pupal elytra gap.

### Knockdown of Two Putative *O*-Fucosyltransferases Showed Entirely Different Effects

The red flour beetle genome has two putative *O-*fucosyltransferases: *Ofut1*, which transfers Fuc specifically to *O-*glycosylation sites in the EGF domain of secreted proteins (including Notch, Delta, and Serrate), and *Ofut2*, which is responsible for *O-*fucosylation of thrombospondin type I repeats (TSRs) in proteins (Ten Hagen et al., 2009; Figure 1). While silencing of *TcOfut1* caused severe effects on pupa and adult development, RNAi of *TcOfut2* did not cause any phenotypical effect (Figure 4). Injection of dsRNAs targeting *TcOfut1* resulted in a significant increase in the gap between the pupal elytra (88% compared to control, *P* = 0.017) and a decrease of the pupal elytra length (5%, *P* < 0.001).

### RNAi of *Rumi* and Fng Caused Minor Effects

Silencing of the transcription levels for *Tcfng* and *TcRumi*, which are orthologs of the *Drosophila* Fuc-specific *O-*GlcNAc transferase (*fng*) and protein *O-*glucosyltransferase (Rumi), caused minor effects. Fng is responsible for transferring GlcNAc specifically to *O-*Fuc residues on the Notch protein (Moloney et al., 2000). The function of Rumi is adding Glc to serine residues in certain EGF repeats of Notch. RNAi of *TcRumi* resulted in a significant reduction of the pupal elytra gap by 32% (*P* = 0.032) as shown in Supplementary Table 3. Knockdown of *Tcfng* decreased the pupal elytra gap by 40% (*P* < 0.001). No appreciable phenotypes were observed after injecting specific dsRNA for these genes to larvae.

### RNAi of *TcGALE1* Reduced Adult Mobility

**FIGURE 6:**
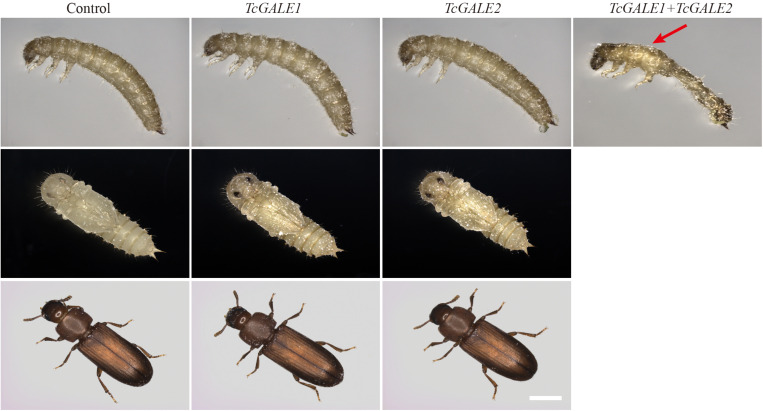
Silencing of GALE genes blocks pupation. Individual RNAi of *TcGALE1* or *TcGALE2* caused minor effects, Silencing of *TcGALE1* and *TcGALE2* simultaneously resulted in mortality at the prepupal stage. Red arrow indicates the split of dorsal cuticle. Scale bar is 1 mm.

*TcGALE1* and *TcGALE2* are two putative genes encoding UDP-galactose 4-epimerases. These two enzymes catalyze two distinct but similar reactions: the epimerization of UDP-Glc to UDP-Gal, and the epimerization of UDP-GlcNAc to UDP-GalNAc. These nucleotide-activated sugars can function as sugar donors in *O-*glycosylation. Therefore, we assumed that knockdown of *GALE* can significantly reduce the related glycans simultaneously. To verify this assumption, we injected specific dsRNAs targeting either of the two GALEs into 4^th^-instar larvae. The induced RNAi effects persisted throughout the development and into the adult stage, and resulted in an approximately 75% reduction of transcript abundance (Supplementary Figure 2). Knockdown of either *TcGALE1* or *TcGALE2* caused no effects on morphology (Figure 6). However, silencing of *TcGALE1* expression resulted in a decrease in the mobility of the adult insect (Figure 5), since the movement speed showed a 13% decrease compared to the control insect (*P* = 0.028).

### Single, Double or Triple RNAi Yield Similar Phenotypes

In addition to the experiments where only one of the *O-*glycosylation pathways was silenced, double and triple RNAi experiments were conducted to assess the redundancy of genes involved in the same pathway and the effect of blocking multiple *O-*glycosylation pathways on the development and survival of the insects. Several combinations were selected: Ofut1, EOGT and Rumi are enzymes known to transfer *O-*glycans to the EGF repeats on the extracellular domain of secreted proteins in insects, and silencing of *TcOGT*, *TcPOMT1* and *TcPOMT2* simultaneously could block all the intracellular *O-*glycans, except for mucin (*O-*GalNAc)-type *O-*glycans. While co*-*silencing usually revealed a phenotype similar to that of the silencing of a single gene, the co-silencing of GALE1 and GALE2 showed additive effects. Co-silencing of *TcGALE1* and *TcGALE2* simultaneously resulted in a 77%-increase in lethality before the pupal stage (Table 3). The insects that died showed a kraurotic abdomen and a split of the dorsal cuticle (Figure 6). This phenotype was similar to the one caused by RNAi of Tcpgant35A. Injection of dsRNAs targeting *TcOGT-TcEOGT*, *Tcpgant3-TcOGT*, *Tcpgant35A-TcOGT*, and *TcOGT-TcPOMT1-TcPOMT2* caused similar phenotypes (Supplementary Figure 3). In all cases, a decrease in pupal elytra size was observed together with a misorientation of the antennae in the pupa and adult insects. At the adult stage, the insects additionally showed a bending of the body and these were unable to close their wings (Supplementary Figure 3). Also for RNAi of *Tcpgant3-TcEOGT* and *TcOfut1-TcEOGT-TcRumi* similar phenotypes were observed, co-silencing of these genes caused an increase in the elytra gap, a bending of the adult body, and a misorientation of the antennae (Supplementary Figure 3). Co-silencing of *Tcpgant5* and *TcOGT* impaired adult wing formation and decreased larval growth as well as pupal elytra length (8%, P < 0.001). RNAi of *Tcpgant3*-*Tcpgant5-Tcpgant35A* caused pupa and adult elytra to be apart (Supplementary Figure 3). Double RNAi of *TcOfut1* and *TcOfut2* resulted in the malformation of the adult elytra (Supplementary Figure 3). RNAi of *TcPOMT1* and *TcPOMT2* caused malformation of the adult wing and a reduction in length of pupal elytra (5%, *P* = 0.011). In most cases, double or triple RNAi resulted in partial larval mortality and partial adult mortality (Table 3).

**TABLE 3:** Summary of phenotypes caused by double and triple RNAi.

Gene	Mortality(%)			Phenotypes caused by RNAi
	larva	pupa	adult	
*TcGALE1* + *TcGALE2*	77.0^a^	3.5	5.4	Pupation blocked
*TcEOGT* + *TcOGT*	30.7	0.0	39.0	Pupal elytra shorter Antenna misorientation Adult wing apart Adult body bent
*Tcpgant3* + *TcEOGT*	14.7	0.0	22.3	Pupal elytra shorter Adult elytra apart Adult body bent Antenna misorientation
*TcPOMT1* + *TcPOMT2*	20.0	0.0	45.8	Pupal elytra shorter Adult elytra malformed
*Tcpgant3* + *TcOGT*	10.0	0.0	35.0	Antenna misorientation Pupal elytra shorter Adult wing apart Adult body bent
*TcOfut1* + *TcOfut2*	11.7	0.0	7.5	Adult elytra malformed
*Tcpgant5* + *TcOGT*	33.3	0.0	20.0	Pupal elytra shorter Adult elytra malformed
*Tcpgant35A* + *TcOGT*	24.0	1.7	43.7	Pupal elytra shorter Antenna misorientation Adult wing apart Adult body bent
*TcOfut1* + *TcEOGT* + *TcRumi*	21.7	1.7	26.7	Antenna misorientation Adult wing apart Adult body bent
*TcOGT* + *TcPOMT1* + *TcPOMT2*	35.3	2.2	15.8	Pupal elytra shorter Antenna misorientation Adult wing apart Adult body bent
*Tcpgant3* + *Tcpgant5* + *Tcpgant35A*	42.2	2.4	8.4	Pupal elytra apart Adult wing apart

*^*a*^Majority of insects died during transition from larva to pupa.*

## Discussion

### Identification and Expression Analysis of OGRGs in *T. castaneum*

A bioinformatics study resulted in the identification of 28 putative OGRGs in the genome of *T. castaneum*. To investigate the role of *O-*glycosylation in the post-embryonic development of insects, we studied the transcription profiles of 19 of the OGRGs in the red flour beetle during its development from egg to adult and in different tissues (including head, thorax, abdomen, wings and legs) at the pupa and adult stage of the red flour beetle. As observed in *D. melanogaster* (Hagen et al., 2003), the overall temporal expression profile showed the highest expression at the pupal stage and a higher expression at the adult stage compared to the larval stage. This profile is similar to the one observed for *N-*glycosylation related genes (NGRGs) in *T. castaneum* (Walski et al., 2016). Their increase in expression levels at the onset of metamorphosis suggested a role for *N-*glycosylation in the transition from larva to adult. Similarly, we hypothesized a role for the OGRGs in the post-embryonic development of insects.

The spatial profile revealed that most of the OGRGs were highly expressed in the wings both at the pupal and adult stage, suggesting that *O-*glycosylation is required for wing development. This is consistent with previous studies in *Drosophila* showing several OGRGs with a strong expression in the wing disc of the third-instar larvae (Tian and Hagen, 2006) (Gelbart and Emmert, 2013).

### Functional Analysis of OGRGs During Post-embryonic Development of *T. castaneum*

**FIGURE 7:**
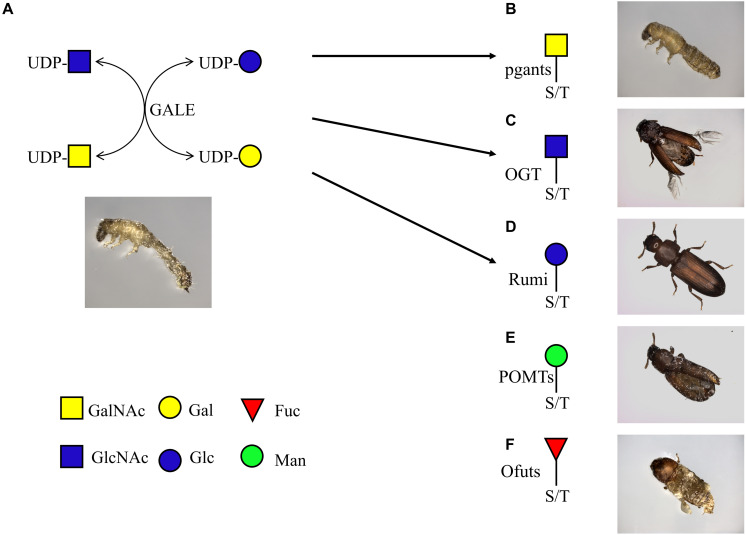
Overview of rnai effects of *O-*glycosylation-related genes in the post-embryonic development of the red flour beetle *Tribolium castaneum*. **(A)** Break of the interconversion of UDP-GalNAc and UDP-Gal, UDP-GlcNAc and UDP-Glc blocked pupation. **(B)** RNAi of N-acetylgalactosaminyltransferases impaired pupation and eclosion. **(C)** Knockdown of *O-*GlcNAc transferases caused malformation of wing and antenna. **(D)** RNAi of protein *O-*mannosyltransferases resulted in malformation of adult wing. **(E)** Silencing of protein *O-*glucosyltransferase caused minor effects. **(F)** Knockdown of *O-*fucosyltransferases conduced to disruption of adult wing development.

To investigate the role of *O-*glycosylation in the metamorphosis and wing development of *T. castaneum*, we silenced the expression of the OGRG through RNAi. The results showed a broad range of phenotypes from blockage of pupation and high mortality to more mild effects on the development of wings and adult mobility (Figure 7). While many phenotypes were consistent with previous silencing and mutation experiments in *D. melanogaster* (reviewed in Li et al., 2020) and *T. castaneum* (Dönitz et al., 2015), several differences were observed with the previously described phenotypes. Although the non-lethal phenotype after silencing of *Tcpgant3* is consistent with the observations in *D. melanogaster* and mutations in *T. castaneum* (Zhang et al., 2008; Dönitz et al., 2015; Yamamoto-Hino et al., 2015), differences were observed with the results in the iBeetle-database of *Tribolium* RNAi phenotypes (Dönitz et al., 2015). While in our hands silencing of *Tcpgant3* only causes a slight reduction in adult motility, iBeetle reports partial mortality (up to 50%) and a delay or block of eclosion (Dönitz et al., 2015). These differences with the RNAi experiments in the iBeetle database could be related to the small sample size of the experiments in iBeetle where only 10 insects were injected for each target gene. The phenotypes of the other OGRGs in the synthesis of mucin-type *O-*glycans are consistent with the results in *D. melanogaster*, with RNAi of *Tcpgant5* and *Tcpgant35A* causing severe lethality (Tran et al., 2012; Yamamoto-Hino et al., 2015) and silencing of *C1GalTA* causing a potential defect in the nervous system (Lin et al., 2008; Yoshida et al., 2008). Interesting, knock-down of *pgant35A* transcription levels resulted in major lethality during the transition from larva to pupa and from pupa to adult, with the insects that died between larva and pupa showing a split in the dorsal cuticle and the beetles that died just before the adult stage showing a cephalothorax in adult appearance while their abdomen was in pupal appearance. This indicated that in the RNAi insects pupation and eclosion were initiated but could not be completed.

While the major lethality (about 80%) and decreased larval growth, observed upon silencing of *OGT* are consistent with previous studies in *D. melanogaster* (Sinclair et al., 2009; Park et al., 2011), silencing in *T. castaneum* also caused a strong phenotype on wings, antennae and body, which is in agreement with the data from iBeetle-Base (Dönitz et al., 2015), being described as bent antennae and irregular wing orientation. However, some differences in the timing of the phenotype were observed for RNAi of *TcEOGT* compared to *D. melanogaster*. The pupal wing blistering in *T. castaneum* was also observed in *D. melanogaster* but at adult stage and at a high rate (Müller et al., 2013). In addition, loss of *EOGT* caused larval lethality in fruit flies but resulted in adult lethality in the red flour beetle (Cox and Spradling, 2006; Buszczak et al., 2007; Neely et al., 2010; Leonardi et al., 2011; Müller et al., 2013). Most of these beetles could not finish their molting completely, suggesting a novel role for *EOGT* during eclosion.

In contrast with the twisted abdomen observed in fruit flies with a mutant *POMT* (Ichimiya et al., 2004), RNAi-induced silencing of *POMT* in *T. castaneum* caused a malformation of the adult wings, suggesting novel roles for *O-*mannosylation in Coleoptera. In addition, RNAi of the POMTs in *Tribolium* caused major lethality (about 65%), whereas complete lethality and behavior defective phenotypes (flight and locomotor behavior defective) were reported in the fruit fly (Ichimiya et al., 2004; Ueyama et al., 2010). In agreement with a previous study in *Drosophila* (Ichimiya et al., 2004), RNAi of each or both of the genes resulted in a similar phenotype, suggesting that POMT1 and POMT2 are dependent on each other.

The effects of RNAi-induced silencing of *TcOfut1* and *TcOfut2* showed contrasting results. While RNAi of *TcOfut1* resulted in a severe phenotype with disrupted wing development and high mortality, no phenotypes were observed after injecting dsRNAs specific to *TcOfut2*. The potential reason for this could be the different targets for the two enzymes (Luo et al., 2006).

Previous studies in *Drosophila* (Ishio et al., 2015; Matsumoto et al., 2016) suggested a redundant function for Ofut1 and Rumi, which catalyze the addition of Glc to serine residues in certain EGF repeats of Notch. However, our results of single RNAi showed that RNAi of *TcOfut1* caused severe effects, while RNAi of *TcRumi* had a minor impact. Specifically, while the injection of dsOfut1 caused a malformation of the elytra and a high adult mortality, we only observed a smaller pupal elytra gap upon silencing of Rumi, which indicated a unidirectional complementation of Ofut1 to Rumi in *T. castaneum*. Combining the results of double RNAi, we expect that Ofut2 plays a role in controlling the complementation between Ofut1 and Rumi. Co-silencing Ofut2 and Ofut1 activated the complementation of Rumi to Ofut1. That could be an explanation for the low mortality in double RNAi of *TcOfut1* and *TcOfut2*.

Interestingly, we did not observe any clear RNAi phenotypes and less than 10% mortality after the larval injection of *dsTcfng*, whereas in the iBeetle-Base, pupal injection resulted in 90% mortality and blocked eclosion (Dönitz et al., 2015). A reason for this discrepancy could be found in the differences in experimental setup. In the iBeetle-Base experiments, female pupa were injected with the *dsTcfng* (Dönitz et al., 2015), while in our experiments 4th instar larvae were treated. This emphasizes the need for comparative research to understand these differences in phenotypes after silencing the same gene but in different developmental stages.

Disruption of GALE, the enzyme catalyzing the interconversion of UDP-Gal and UDP-Glc, and UDP-GalNAc and UDP-GlcNAc, affects multiple *O-*glycosylation pathways as it causes a dependency not only on exogenous Gal but also on exogenous GalNAc being a necessary precursor for the synthesis of *O-*glycans. The high larval mortality when silencing the expression of *TcGALE*, is consistent with previous studies showing embryonic and larval lethality in *D. melanogaster* (Sanders et al., 2010; Daenzer et al., 2012). In humans, mutations in GALE result in epimerase-deficiency galactosemia, a disease characterized by liver damage, early-onset cataracts, deafness and cognitive disability (Timson, 2006). Interestingly, the two putative genes of *GALE* (*TcGALE1*, *TcGALE2*) identified in the genome of *T. castaneum*, showed an opposite expression pattern. Whereas RNAi of either *TcGALE1* or *TcGALE2* caused almost no effects on viability, co-silencing of both *TcGALE1* and *TcGALE2* significantly reduced the viability at the prepupal stage. This suggests that the proteins coded by these two genes have, at least partially overlapping functions. In addition, the split on the dorsal cuticle indicates that pupation was initiated but not completed, suggesting that GALE or *O-*glycans consisting out of these four sugars play an important role in pupation.

The *O-*glycosylation is complicated and diverse. For example, the insect genome encodes more than 12 *pgant* genes, that are functionally redundant in transferring GalNAc to proteins. In this case, double or triple RNAi could be a more powerful strategy to study gene function. However, from an overall perspective, most double and triple RNAi experiments resulted in almost identical effects compared to single RNAi. This was against our expectations. When performing the double or triple RNAi experiments, we injected the same amount of the individual dsRNAs as we did in the single RNAi experiments and similar RNAi efficiencies were obtained for each gene in the triple RNAi experiments as in the single RNAi experiments. The results observed in the triple RNAi experiments are consistent over independent biological repeats. A reasonable explanation for the phenotypes observed in the triple RNAi of the pgants could be that these pgants or their targets probably function in a particular order in biological processes. For instance, if *Pgant5* plays a role upstream in the biological processes, then silencing of *Tcpgant5* already disrupted the pathway, and consequently, this triple RNAi showed a similar phenotype as RNAi of *Tcpgant5*. Another possible explanation is that due to the increased pressure by silencing three genes a compensation effect takes place. A similar observation was made when silencing the catalytic subunit of the *N-*glycosylation oligosaccharyl transferase complex, after silencing of one isoform, the expression of the other isoform increased (De Schutter et al., 2019). Nevertheless, it is interesting that all double or triple RNAi experiments involving *TcOGT* resulted in a significantly higher larval mortality than the single RNAi, while the larval mortality was only minor in the single RNAi of *TcOGT*. In contrast, RNAi of *Tcpgant35A* and *TcOGT* caused 22% lower larval mortality than the single RNAi of *Tcpgant35A*. These results indicate a complicated interplay between different OGRGs.

In conclusion, we demonstrated that *O-*glycosylation is essential for survival, metamorphosis and wing development of the red flour beetle, *T. castaneum*. Moreover, we report that the core 1 *O-*glycan synthesized by pgant35A is required for pupation and eclosion. Our results provide an insight into the functions of *O-*glycosylation during the post-embryonic development of insects.

## Data Availability Statement

The datasets presented in this study can be found in online repositories. The names of the repository/repositories and accession number(s) can be found in the article/Supplementary Material.

## Author Contributions

WL performed the gene expression analysis, RNAi experiments, and data analyses. KDS and WL performed the phylogenetic analysis. EVD, GS, and KDS were involved in data analysis and discussion and manuscript corrections. All authors contributed to manuscript and approved the final manuscript.

## Conflict of Interest

The authors declare that the research was conducted in the absence of any commercial or financial relationships that could be construed as a potential conflict of interest.

## Footnotes

1 http://ibeetle-base.uni-goettingen.de/blast/

2 https://www.ncbi.nlm.nih.gov/guide/proteins/

3 https://www.ebi.ac.uk/interpro/download/

4 https://blast.ncbi.nlm.nih.gov

5 http://www.bioinformatics.nl/cgi-bin/primer3plus/primer3plus.cgi

6 https://fiji.sc/

## Supplementary Material

Supplementary Figure 1 Phylogeny of putative proteins involved in *O-*glycosylation in *Tribolium castaneum*. Phylogenetic tree was constructed in MEGA X using Maximum Likelihood method. The best fitting model LG + G **(A–E,G)** or WAG + F + I **(F,H,I)** was determined based on the BIC score. The phylogeny was tested with 1000 bootstrap replicates. Bootstrap values are indicated at the branches. Hs, *Homo sapiens*; Dm, *Drosophila melanogaster;* Tc, *Tribolium castaneum*; Nl, brown planthopper (*Nilaparvata lugens*); Ld, colorado potato beetle (*Leptinotarsa decemlineata*); Bt, large earth bumblebee (*Bombus terrestris*); Ap, pea aphid (*Acyrthosiphon pisum*); Bm, silk moth (*Bombyx mori*). **(A)** GALE orthologs. **(B)** C1GalT orthologs. **(C)** OGT and EOGT orthologs. **(D)** Ofut orthologs. **(E)** POMT orthologs. **(F)** orthologs of fng and Rumi. **(G)** pgant orthologs. **(H)** orthologs of proteins involved in synthesis of heparan sulfate and chondroitin sulfate. **(I)** all OGRG orthologs from **(A–H)**.

Supplementary Figure 2 Efficiency of gene silencing. Fourth-instar larvae were injected with approx. 200 ng dsRNA. DsGFP was injected in control insects. Total RNA was extracted at 6 d (larva), 11 d (pupa), 16 d (adult) after injection. Relative gene expression was measured by qPCR using Ubiquitin and RPS18 as reference genes. Error bar is SD (*n* = 2). ^∗^, the gene expression was not examined because of high mortality.

Supplementary Figure 3 Phenotypes caused by double and triple RNAi in *Tribolium castaneum*. In the double and triple RNAi experiments, fourth-instar larvae were injected with a same amount of dsRNA for each gene as in the single RNAi experiments. DsGFP was injected in the control. Scale bar is 1 mm.

Supplementary Table 1 Primers used for gene expression analysis.

Supplementary Table 2 Primers used for dsRNA synthesis. taatacgactcactatagggaga – T7 promoter sequence.

Supplementary Table 3 Pupal elytra length and gap after dsRNA injection at larva stage.

Supplementary Table 4 List of all identified genes.

Supplementary Table 5 Gene silencing efficiency of double and triple RNAi.
