# Supplementary figures and images for: RNAi-Mediated Silencing of Pgants Shows Core 1 O-Glycans Are Required for Pupation in Tribolium castaneum
Source: Front Physiol. 2021 Mar 24;12:629682. doi: 10.3389/fphys.2021.629682 (PMC8024498; doi:10.3389/fphys.2021.629682)

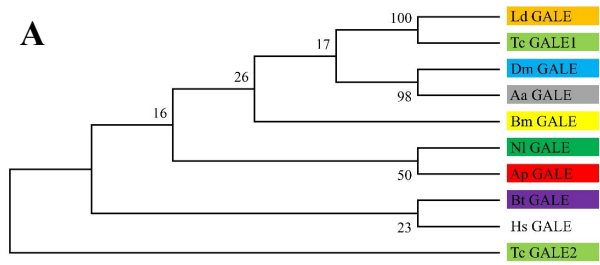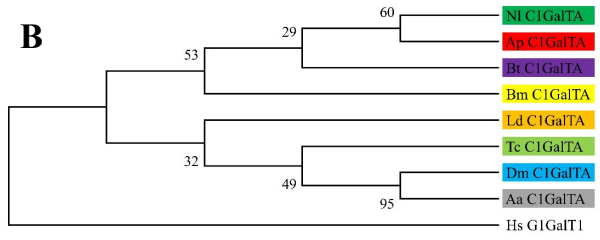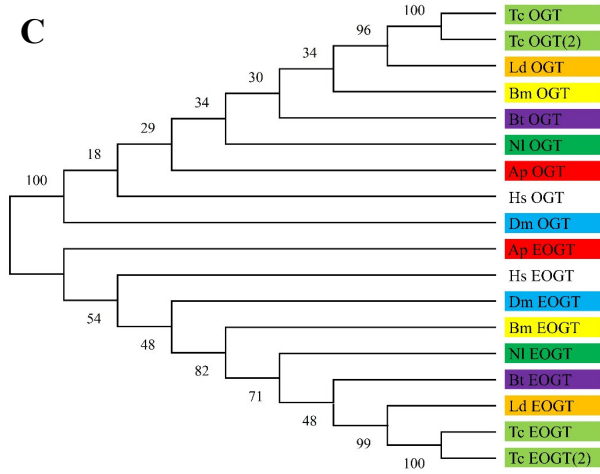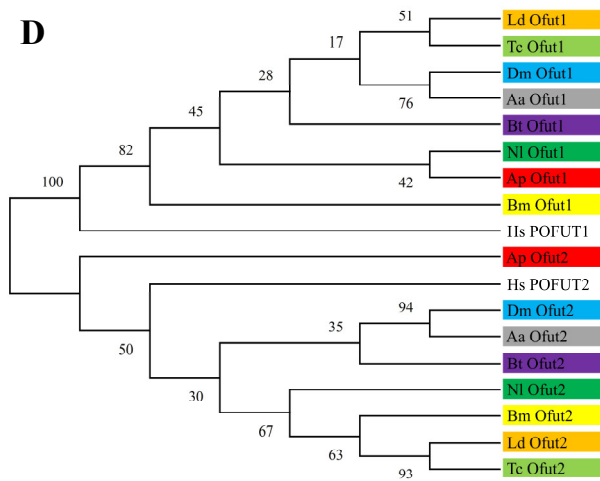

**E**

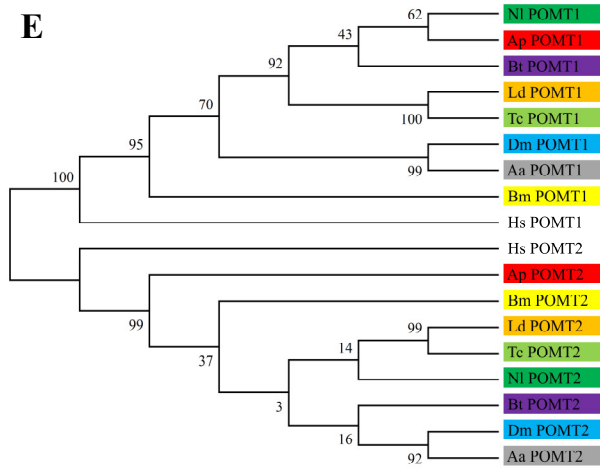

**F**

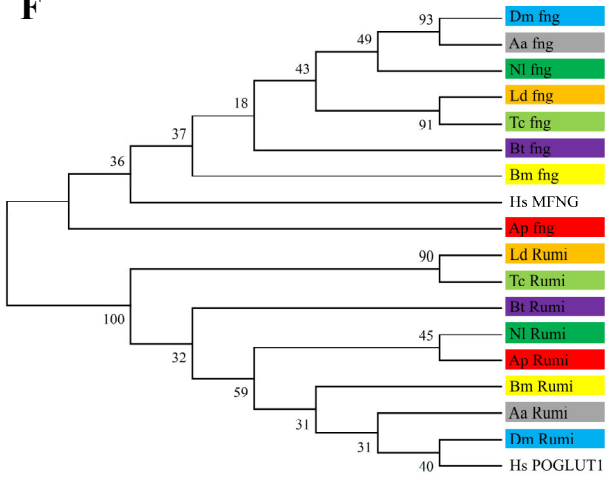

Supplement: Supplementary Figure 1 — Phylogeny of putative proteins involved in O-glycosylation in Tribolium castaneum. Phylogenetic tree was constructed in MEGA X using Maximum Likelihood method. The best fitting model LG + G (A–E,G) or WAG + F + I (F,H,I) was determined based on the BIC score. The phylogeny was tested with 1000 bootstrap replicates. Bootstrap values are indicated at the branches. Hs, Homo sapiens; Dm, Drosophila melanogaster; Tc, Tribolium castaneum; Nl, brown planthopper (Nilaparvata lugens); Ld, colorado potato beetle (Leptinotarsa decemlineata); Bt, large earth bumblebee (Bombus terrestris); Ap, pea aphid (Acyrthosiphon pisum); Bm, silk moth (Bombyx mori). (A) GALE orthologs. (B) C1GalT orthologs. (C) OGT and EOGT orthologs. (D) Ofut orthologs. (E) POMT orthologs. (F) orthologs of fng and Rumi. (G) pgant orthologs. (H) orthologs of proteins involved in synthesis of heparan sulfate and chondroitin sulfate. (I) all OGRG orthologs from (A–H). [file Image_1.pdf]
