# Supplementary material for: RNAi-Mediated Silencing of Pgants Shows Core 1 O-Glycans Are Required for Pupation in Tribolium castaneum
Source: Front Physiol. 2021 Mar 24;12:629682. doi: 10.3389/fphys.2021.629682 (PMC8024498; doi:10.3389/fphys.2021.629682)
Supplement: Supplementary Table 1 — Primers used for gene expression analysis. [file Table_1.docx]

**Supplementary TABLE S1. Primers used for gene expression analysis.**

| **Gene** | **Primer sequence (5’ to 3’)**  **(forward primers are listed first)** | **Amplicon**  **length** | **Amplification Efficiency (%)** |
| --- | --- | --- | --- |
| *Tcpgant1* | GGTGGGGAGAATCTGGAAAT  TGTCCTTGTTATCCGGGAAG | 130 | 102 |
| *Tcpgant2* | CTGAGGACGGAGAGGAGTTG  GCACCCTCGATCTCATCAAT | 87 | 97.5 |
| *Tcpgant3* | GTCGTTCAGGATCTGGCAAT  GGTTCGAGAAAAGCGTCTTG | 128 | 92.5 |
| *Tcpgant5* | GGGTTCGTACGACGAAGGTA  CCAGGAAACGTGTACGGACT | 150 | 101.5 |
| *Tcpgant6* | GTCATGGCTGGAGGTTTGTT  GCCTCCACACTGCCATATTT | 129 | 97.9 |
| *Tcpgant7* | AGTCCACTCGGTCATCAACC  GGTTCTCCTTGTCGCTGAAG | 83 | 90.1 |
| *Tcpgant9* | CGCGAAGGTTTGATTAGAGC  CCTTGCTATCCGATCCAAAA | 129 | 93.0 |
| *Tcpgant35A* | TGAATGTTGGCTGGATTGAA  TGAAGCCTCCCCTGACTAGA | 135 | 93.0 |
| *TcC1GalTA* | GCTGATTGGTTCCTCAAAGC  CCGCTCATGTAACCCTGTTT | 143 | 97.9 |
| *TcOGT* | ACGCCAGGATGAAGAAATTG  ACGGAACTCGTGTGATAGGG | 113 | 94.8 |
| *TcEOGT* | GCATGATTTTCGGGCTGTAT  TGAAAGGGGATTTTGAGTCG | 112 | 106.5 |
| *TcOfut1* | ACCGGCTTTTCTTCTTGGTT  ACCCAGAGAGCCCAAAAAGT | 144 | 91.4 |
| *TcOfut2* | GCCCTATTTAGGGGTGCATT  GGCGATAAAAACCTGCGATA | 136 | 96.5 |
| *Tcfng* | ACCAGTTCCACTCGCATCTC  TCGAATCCCTCGATCTTGAC | 124 | 91.3 |
| *TcPOMT1* | ATTCACCGGACGACAACTCC  TTCGCGTTCGCGTTCTTTTT | 145 | 106.8 |
| *TcPOMT2* | GCAGTCAGCAGCAAATTGAA  CTCTGAGATTGGGGTTGCAT | 124 | 92.8 |
| *TcRumi* | GGCTGCCAGTTTTCGTTTTA  CGCCTCCACTGGAATGTAAT | 127 | 100.0 |
| *TcGALE1* | CCGGTTACGTAGGCTCTCAC  GGACTCCGGTTTTTGGTTTT | 113 | 95.3 |
| *TcGALE2* | GAACATCTTCGCAAAGCACA  TGTTCCATGATCTCGAGCAG | 141 | 92.1 |
| *TcRPS18* | ACGCAAAGTCATGTTTGCCC  TTCGTCGGAACACTCACCAG | 118 | 94.0 |
| *TcUbiquitin* | AGATCCTCCCGCACAATGTT  GGCTTGCCAGTGAAATAAATCAT | 61 | 92.6 |
