# Supplementary material for: RNAi-Mediated Silencing of Pgants Shows Core 1 O-Glycans Are Required for Pupation in Tribolium castaneum
Source: Front Physiol. 2021 Mar 24;12:629682. doi: 10.3389/fphys.2021.629682 (PMC8024498; doi:10.3389/fphys.2021.629682)
Supplement: Supplementary Table 2 — Primers used for dsRNA synthesis. taatacgactcactatagggaga – T7 promoter sequence. [file Table_2.docx]

Supplementary **TABLE S2. Primers used for dsRNA synthesis.**

| **Gene** | **T7+Primer sequence (5’ to 3’)**  **(forward primers are listed first)** | **Amplicon**  **length** |
| --- | --- | --- |
| *Tcpgant3* | taatacgactcactatagggagaCAAGACGCTTTTCTCGAACC | 499 |
|  | taatacgactcactatagggagaTTCCTGCTTCTCCTTGTCGT |  |
| *Tcpgant5* | taatacgactcactatagggagaACGTCGTCATCCTTTCGTTC | 453 |
|  | taatacgactcactatagggagaCACTCCAAACGGTCCTCAAT |  |
| *Tcpgant35A* | taatacgactcactatagggagaTCAGAGTCGCAAATGTCTGG | 550 |
|  | taatacgactcactatagggagaCATGTGCCTGCAGCTATGTT |  |
| *TcC1GalTA* | taatacgactcactatagggagaTGGTCGAACGTAAACAAGCA | 457 |
|  | taatacgactcactatagggagaGCATGTGGTTGTGGTAGACG |  |
| *TcOGT* | taatacgactcactatagggagaAACCCCCATTGAAAACATGA | 403 |
|  | taatacgactcactatagggagaCAACTGTCCCCTCCTCACAT |  |
| *TcEOGT* | taatacgactcactatagggagaAACCTCCCCGATTCTCACTT | 426 |
|  | taatacgactcactatagggagaGCCGTCTTTTAAAACGTCCA |  |
| *TcOfut1* | taatacgactcactatagggagaCGCCCCCTTCACAAATACCT | 485 |
|  | taatacgactcactatagggagaTTTCCCGCTTGACAAAGGC |  |
| *TcOfut2* | taatacgactcactatagggagaTGCAACTGCAGCCAGGATTA | 565 |
|  | taatacgactcactatagggagaTTTTGGCGGACGATTTGAGC |  |
| *Tcfng* | taatacgactcactatagggagaACCTGGTTTTTCACCGACAC | 472 |
|  | taatacgactcactatagggagaTCAGCAGGTGCTCGATTATG |  |
| *TcPOMT1* | taatacgactcactatagggagaCGTTTCGTCCCTGCGTTTTT | 504 |
|  | taatacgactcactatagggagaATCATGTGGTCCCGCTTTGT |  |
| *TcPOMT2* | taatacgactcactatagggagaTTGGTGGGCCAATATCATTT | 444 |
|  | taatacgactcactatagggagaCACGTGTCCAACCATTTCAG |  |
| *TcRumi* | taatacgactcactatagggagaTGCAACCACAATAGTGCGAG | 499 |
|  | taatacgactcactatagggagaCGGGAACATGCAGTTCTTGTC |  |
| *TcGALE1* | taatacgactcactatagggagaGTCCAGGTGCCCCTAATGTA | 434 |
|  | taatacgactcactatagggagaTAGTCACGGACTCCCGTACC |  |
| *TcGALE2* | taatacgactcactatagggagaGTTTACGGCGAACCCACTTA  taatacgactcactatagggagaGTACTACGGTGCCGGTGACT | 481 |

taatacgactcactatagggaga – T7 promoter sequence.
