# Supplementary material for: RNAi-Mediated Silencing of Pgants Shows Core 1 O-Glycans Are Required for Pupation in Tribolium castaneum
Source: Front Physiol. 2021 Mar 24;12:629682. doi: 10.3389/fphys.2021.629682 (PMC8024498; doi:10.3389/fphys.2021.629682)
Supplement: Supplementary Table 3 — Pupal elytra length and gap after dsRNA injection at larva stage. [file Table_3.docx]

**Supplementary Table S3. Pupal elytra length and gap after dsRNA injection at larva stage.**

| **Gene of RNAi** | **Elytra length (mm)**  **M ± SE** | | **P value** | **Elytra gap (mm)**  **M ± SE** | | **P value** |
| --- | --- | --- | --- | --- | --- | --- |
|  | **RNAi** | **Control** |  | **RNAi** | **Control** |  |
| *Tcpgant3* | 1.52±0.011 | 1.53±0.003 | 0.257 | 0.07±0.001 | 0.06±0.005 | 0.736 |
| *Tcpgant5* | 1.50±0.013 | 1.52±0.011 | 0.080 | 0.08±0.002 | 0.06±0.011 | 0.007 |
| *Tcpgant35A* | 1.32±0.007 | 1.52±0.011 | 0.000 | 0.10±0.009 | 0.06±0.011 | 0.045 |
| *TcC1GalTA* | 1.55±0.006 | 1.54±0.002 | 0.805 | 0.05±0.005 | 0.04±0.013 | 0.914 |
| *TcOGT* | 1.39±0.004 | 1.54±0.002 | 0.000 | 0.24±0.029 | 0.04±0.013 | 0.000 |
| *TcEOGT* | 1.51±0.002 | 1.52±0.001 | 0.598 | 0.11±0.011 | 0.07±0.002 | 0.014 |
| *TcOFut1* | 1.45±0.080 | 1.52±0.021 | 0.000 | 0.15±0.079 | 0.08±0.012 | 0.017 |
| *TcOFut2* | 1.55±0.006 | 1.52±0.021 | 0.079 | 0.07±0.022 | 0.08±0.012 | 0.226 |
| *Tcfng* | 1.53±0.004 | 1.52±0.001 | 0.898 | 0.06±0.022 | 0.10±0.027 | 0.000 |
| *TcPOMT1* | 1.40±0.002 | 1.56±0.008 | 0.000 | 0.08±0.020 | 0.08±0.012 | 0.753 |
| *TcPOMT2* | 1.42±0.049 | 1.56±0.008 | 0.000 | 0.06±0.012 | 0.08±0.012 | 0.152 |
| *TcRumi* | 1.55±0.007 | 1.52±0.001 | 0.030 | 0.05±0.005 | 0.07±0.002 | 0.032 |
| *TcGALE1* | 1.52±0.003 | 1.53±0.021 | 0.078 | 0.07±0.014 | 0.07±0.017 | 0.459 |
| *TcGALE2* | 1.53±0.029 | 1.53±0.021 | 0.665 | 0.08±0.027 | 0.07±0.017 | 0.422 |
