# Supplementary material for: RNAi-Mediated Silencing of Pgants Shows Core 1 O-Glycans Are Required for Pupation in Tribolium castaneum
Source: Front Physiol. 2021 Mar 24;12:629682. doi: 10.3389/fphys.2021.629682 (PMC8024498; doi:10.3389/fphys.2021.629682)
Supplement: Supplementary Table 4 — List of all identified genes. [file Table_4.docx]

Supplementary **Table S4. List of all identified genes.**

| ***Drosophila***  **gene** | ***Tribolium* orthologs** | **Function** |
| --- | --- | --- |
| *pgant1* | TC015209 | polypeptide GalNAc transferase |
| *pgant2* | TC007223 | polypeptide GalNAc transferase |
| *pgant3* | TC005702 | polypeptide GalNAc transferase |
| *pgant4* | TC008338 | polypeptide GalNAc transferase |
| *pgant5* | TC005902 | polypeptide GalNAc transferase |
| *pgant6* | TC008338 | polypeptide GalNAc transferase |
| *pgant7* | TC008465 | polypeptide GalNAc transferase |
| *pgant8* | TC008338 | polypeptide GalNAc transferase |
| *pgant9* | TC005479 | polypeptide GalNAc transferase |
| *pgant10* | TC008338 | polypeptide GalNAc transferase |
| *pgant11* | TC008338 | polypeptide GalNAc transferase |
| *pgant12* | TC005479 | polypeptide GalNAc transferase |
| *pgant35A* | TC013148 | polypeptide GalNAc transferase |
| *C1GalTA* | TC030587 | Core 1 Galactosyltransferase |
| *OGT (sxc)* | TC003916 | *O-*GlcNAc transferase |
| *EOGT* | TC013339 | EGF domain-specific *O-*GlcNAc transferase |
| *Ofut1* | TC013385 | *O-*fucosyltransferase |
| *Ofut2* | TC030583 | *O-*fucosyltransferase |
| *fng(fringe)* | TC011785 | Fucose-specific *O-*GlcNAc transferase |
| *POMT1 (rt)* | TC010429 | protein *O-*mannosyltransferase |
| *POMT2 (tw)* | TC033711 | protein *O-*mannosyltransferase |
| *Rumi* | TC013552 | protein *O-*glucosyltransferase |
| *GALE* | TC009598 | UDP-galactose-4-epimerase |
|  | TC009301 |  |
| GlcAT-I | TC007027 | Galactosylgalactosylxylosylprotein 3-beta-glucuronosyltransferase |
| GlcAT-S | TC004674 | Galactosylgalactosylxylosylprotein 3-beta-glucuronosyltransferase |
| OXT | TC002371 | Peptide *O-*xylosyltransferase |
| GalTI | TC012167 | Xylosylprotein 4-beta-galactosyltransferase |
| GalTII | TC014260 | beta-1,3-Galactosyltransferase |
| DEXTL3 | TC013377 | Glucuronosyl-galactosyl-proteoglycan 4-alpha-N- acetylglucosaminyltransferase |
| DEXT1 | TC004758 | N-acetylglucosaminyl-proteoglycan 4-beta-glucuronosyltransferase |
| DEXT2 | TC004910 | N-acetylglucosaminyl-proteoglycan 4-beta-glucuronosyltransferase |
| Hs2st | TC013753 | Heparan sulfate 2-*O-*sulfotransferase |
| Hs6st | TC014815 | Heparan sulfate 6-*O-*sulfotransferase |
