# Supplementary material for: RNAi-Mediated Silencing of Pgants Shows Core 1 O-Glycans Are Required for Pupation in Tribolium castaneum
Source: Front Physiol. 2021 Mar 24;12:629682. doi: 10.3389/fphys.2021.629682 (PMC8024498; doi:10.3389/fphys.2021.629682)
Supplement: Supplementary Table 5 — Gene silencing efficiency of double and triple RNAi. [file Table_5.docx]

**Supplementary Table S5. Gene silencing efficiency of double and triple RNAi.**

| **Injection** | **Gene** | **RNAi efficiency %** | |
| --- | --- | --- | --- |
|  |  | **1st replicate** | **2nd replicate** |
| *TcGALE1*+*TcGALE2* | *TcGALE1* | 87.55 | 82.57 |
|  | *TcGALE2* | 52.03 | 84.65 |
| *TcEOGT*+*TcOGT* | *TcEOGT* | 56.60 | 68.80 |
|  | *TcOGT* | 63.57 | 74.31 |
| *Tcpgant3*+*TcEOGT* | *Tcpgant3* | 62.26 | 53.27 |
|  | *TcEOGT* | 71.67 | 77.99 |
| *TcPOMT1*+*TcPOMT2* | *TcPOMT1* | 69.05 | 90.90 |
|  | *TcPOMT2* | 77.19 | 92.55 |
| *Tcpgant3*+*TcOGT* | *Tcpgant3* | 47.92 | 59.22 |
|  | *TcOGT* | 54.24 | 75.32 |
| *TcOfut1*+*TcOfut2* | *TcOfut1* | 80.81 | 83.15 |
|  | *TcOfut2* | 72.30 | 76.56 |
| *Tcpgant5*+*TcOGT* | *Tcpgant5* | 65.53 | 75.02 |
|  | *TcOGT* | 78.54 | 61.06 |
| *Tcpgant35A*+*TcOGT* | *Tcpgant35A* | 62.90 | 68.01 |
|  | *TcOGT* | 77.91 | 60.94 |
| *TcOfut1*+*TcEOGT*+*TcRumi* | *TcOfut1* | 74.56 | 40.79 |
|  | *TcEOGT* | 69.56 | 57.33 |
|  | *TcRumi* | 53.57 | 57.78 |
| *Tcpgant3*+*Tcpgant5*+*Tcpgant35A* | *Tcpgant3* | 64.31 | 56.65 |
|  | *Tcpgant5* | 81.11 | 70.14 |
|  | *Tcpgant35A* | 78.83 | 85.31 |
| *TcOGT*+*TcPOMT1*+*TcPOMT2* | *TcOGT* | 93.42 | 72.74 |
|  | *TcPOMT1* | 95.65 | 74.42 |
|  | *TcPOMT2* | 97.24 | 86.59 |
